# Knockdown of long non-coding RNA PCAT-1 inhibits myeloma cell growth and drug resistance via p38 and JNK MAPK pathways

**DOI:** 10.7150/jca.35098

**Published:** 2019-10-20

**Authors:** Xianjuan Shen, Pei Shen, Qian Yang, Qingqing Yin, Feng Wang, Hui Cong, Xudong Wang, Shaoqing Ju

**Affiliations:** 1 Laboratory Medicine Center, Affiliated Hospital of Nantong University, #20 Xisi Road, Nantong 226001, JS, P. R. China;; 2 Research Center of Clinical Medicine, Affiliated Hospital of Nantong University, #20 Xisi Road, Nantong 226001, JS, P. R. China.

**Keywords:** long non-coding RNA, multiple myeloma, p38, JNK, drug resistance

## Abstract

**Objective:** Both previous and recent literature showed long non-coding RNAs (lncRNAs) were crucial participants in multiple myeloma (MM) evolution. However, the dynamic regulation and mechanism of lncRNAs in MM progression was not fully understood. This study will explore the expression and effects of prostate cancer-associated ncRNA transcript 1 (PCAT-1) in MM.

**Materials and Methods:** The expression level of PCAT-1 was examined using quantitative real-time PCR in patients with newly diagnosed MM and cell lines. The potential biological effects and molecular mechanisms of PCAT-1 in MM were evaluated using a series of soft agar colony formation assay, CCK-8 assay, cell cycle and apoptosis assay by flow cytometry, protein chip arrays, western blot analysis, immunohistochemistry and nude subcutaneous tumorigenesis model.

**Results:** High expression of PCAT-1 was observed in patients with newly diagnosed MM and cell lines. Over-expressed PCAT-1 enhanced cell division and inhibited apoptosis both in cultured cells and in animal model. Meanwhile, silenced PCAT-1 exerted the opposite function. Additionally, PCAT-1 knockdown sensitized MM cells to bortezomib (Bort). Inhibitor of PCAT-1 combination with Bort exhibited a more effective inhibitory effect on MM cells compared with negative control or Bort alone. Further mechanism exploration via protein chips, Go and KEGG pathway analysis along with immunoblot analysis revealed that PCAT-1 facilitated cell growth and drug resistance via the p38 and JNK MAPK pathways.

**Conclusion:** This study identified a novel lncRNA-associated mechanism underlying MM carcinogenesis, and provided clinicians with a promising therapeutic target in MM.

## Introduction

Multiple myeloma (MM) is a malignancy originated in bone marrow plasma cells, which is characterized by aberrant congregation and infiltration of non-functional plasma cells 1. Despite great efforts have been made in myeloma therapy, including exploration of immunomodulatory drugs, application of proteasome inhibitors and transplantation of autologous stem cells, MM remains incurable 2. The potential mechanism involved in MM evolution is not well characterized. It's therefore urgent to make out the cellular mechanisms and develop new diagnostic biomarkers as well as therapeutic targets for MM.

Though most human genome sequences can be normally transcribed, only a small fraction of them are successfully translated into proteins 3. LncRNAs belong to a specific non-coding RNA with no ability to be translated into protein, and have greater than 200 nucleotides in length. Functional researches demonstrated that lncRNAs were closely associated several tumor phenotypes, such as disorganized cell cycle, inordinate tumor growth, abnormal differentiation, distant metastasis and malignant evolution 4-5. At present, various up-regulated lncRNAs are explored to act as laboratory indexes for predicting survivals of MM patients, such as MALAT1, H19, CCAT1 and so on 6-9.

LncRNA-PCAT-1, short for prostate cancer-associated ncRNA transcript 1, was proved to facilitate growth and reduce apoptosis of prostate cancer cells 10. Meantime, PCAT-1 was shown to be a novel therapeutic target that actively increased cell malignancy in non-small cell lung cancer 11. In addition, PCAT-1 was indicated to be overexpressed in hepatocellular carcinoma (HCC) patients and might serve as a novel prognostic indicator in HCC patients with poor outcomes 12. Our previous study found higher serum PCAT-1 expression in MM patients than healthy controls and might have a guiding role in the auxiliary diagnosis of MM 13, yet the specific mechanism of PCAT-1 in MM was poorly described.

This time, we planned to discuss specific roles of PCAT-1 in MM progression. It was discovered that PCAT-1 was significantly up-regulated in primary cells obtained from MM patients and MM cell lines. Through up-regulating and down-regulating PCAT-1 expression artificially, we found PCAT-1 could facilitate cell proliferation, induce cell cycle arrest, inhibit cell apoptosis and weaken drug sensitivity in cultured MM cells. *In vivo* study also confirmed its carcinogenic role. Notably, we identified p38 and JNK MAPK signaling pathways were involved in the biological functions mediated by PCAT-1. Above results identified a crucial role of PCAT-1 in promoting tumor growth and drug resistance through MAPK signaling pathway in MM.

## Materials and methods

### Patients

A total of 18 clinically confirmed MM patients were inpatients of affiliated hospital of Nantong University from February 2016 to July 2018, including 7 females and 11 males ranging in age from 45 to 80 years with a mean of 65±6 years. These patients were newly diagnosed and non-treated. 7 healthy donors who underwent routine physical examinations during the same period were used as the controls. The age of the healthy group ranged from 38 to 65 years with a mean age of 46±7 years. The diagnosis, stage and risk status of MM were made in accordance with the National Comprehensive Cancer Network (NCCN) (2015 version 3 &2017 version 3) 31. The study was performed under approval of local ethics committee.

### Cell culture

Human MM cell lines (NCI-H929, RIMP 8226 and U266 cells) were cultured with RPMI-1640 medium containing 10% fetal bovine serum (FBS) (Gibco, Gaithersburg, USA), 1% streptomycin-penicillin and 1% glutamine in a incubator with constant temperature (37°C) and CO_2_ concentration (5%).

### RNA extraction and real-time PCR

Trizol reagent (Takara, Dalian, China) was used to isolate cells total RNAs. Serum RNA was extracted from a 400 μl volume of serum samples by RNA extraction kit (Life Technologies, US). The cDNA was obtained by the reverse transcription kit (Thermo Fisher Scientific, US) following 42°C for 60min and 70°C for 5min. All amplification procedures were ran on ABI 7500 detection system (ABI, USA). The PCAT-1 primer sequences were as follows: F5′-GAGAGCTGACATAGGCACCC-3′ and R5′-TCTCCACTGGTGTTCATGGC-3′; GAPDH: F5′-TGATGACATCAAGAAGGTGGTGAAG-3′ and R5′-TCCTTGGAGGCCATGTGGGCCAT-3′. The real-time PCR reactions were repeated 3 times. We used the comparative Ct method to quantify the relative expression.

### Plasmid construction and transfection

LncRNA PCAT-1 vectors, including the empty vector (pcDNA-NC), PCAT-1 over-expressed vector (pcDNA-PCAT-1), PCAT-1 knockdown plasmid (sh-PCAT1) and control (sh-NC) were designed by Genepharm Corporation (Shanghai, China). About 1×10^6^ MM cells were seeded in six-well plates per well. All reactions involving transfection were employing Lipofectamine 3000 Reagent (Invitrogen) following the manufacturer's instructions.

### Immunoblot analysis

Total Protein Lysis Buffer was made form RIPA lysate containing 1% PMSF. About 60 μg total proteins were used for sodium dodecyl sulfate polyacrylamide gel electrophoresis (SDS-PAGE) loading followed by transferring to PVDF membrane. After blocking in 5% skim milk, the PVDF membrane was incubated with primary antibodies and secondary antibodies separately in order. Finally, we visualized protein bands using enhanced chemiluminescence (ECL, Amersham Pharmacia). All results were concluded from three repeated experiments.

### Cell proliferation assay

A density of 3000 MM cells per well were seeded in a 96-well plate. Each well was added 10 μl of CCK-8 reagent. Absorbance (A) value was read at 450 nm (650 nm as reference) after incubating for 2 h. Five repeated wells were designed for each group and the experiment was repeated in triplicate.

### Cell cycle and apoptosis assay

PE Annexin V apoptosis detection kit (BD Pharmingen) and adopting propidium iodide cell cycle detected kits (BD Pharmingen) were applied for apoptotic ratio and cell cycle detection by FACS analysis. We collected data from three repeated experiments.

### Soft agar colony formation assay

MM cells, diluted in RPMI-1640 medium containing 20% FBS, were mixed with 3% low-melting point agarose solution, and seeded at a density of 800 cells/well on 6-well plates. Over 50 cells per colony were counted as a clone after culturing 10 days. All experiments were repeated three times.

### Immunohistochemistry study

Transplanted tumor tissues of nude mice were embedded in paraffin. The specimens were cut into 5 μm thickness sections and then stained with hematoxylin-eosin (H&E) in terms of the routine histopathological examination. Immunostained used primary antibodies against ki67 and caspase-3. The avidin-biotin-peroxidase method was used to localize and quantify target proteins. We achieved our photos under a microscope at 400× or 200× (Olympus, Japan).

### Tumor xenografts

BALB/c nude mice (4 weeks old, female) were raised in a sterile environment in Laboratory Animal Center of Nantong University. We achieved approval of the Animal Care Committee of Nantong University before our experiment. 3×10^6^ U266 cells transfected with pcDNA-NC or pcDNA-PCAT-1 were subcutaneously inoculated into the left flank of the nude mice. We measured and calculated tumor volumes using length × width^2^ × 0.5 every 3 days. Mice were euthanized 35 days after injection. Then xenograft tissues were photographed and immunohistochemical stained.

### Protein chip arrays

U266 cells transfected with pcDNA-PCAT-1 were stored in protein lysis buffer and sent to RayBiotech Corporation (Guangzhou, China). Changes in protein expression were assessed according to manufacturer's protocol of protein chip arrays.

### Statistical analysis

Data were expressed as the mean±SD. Statistics analysis software SPSS 18.0 (SPSS, Inc., Chicago, USA) and Graphpad Prism 6.0 (San Diago, USA) were applied. All experiments were performed three times independently. Statistical significance was calculated using the Student's t-test. P <0.05 was considered as statistically significant.

## Results

### PCAT-1 is up-regulated in MM samples and cells

**Figure 1:**
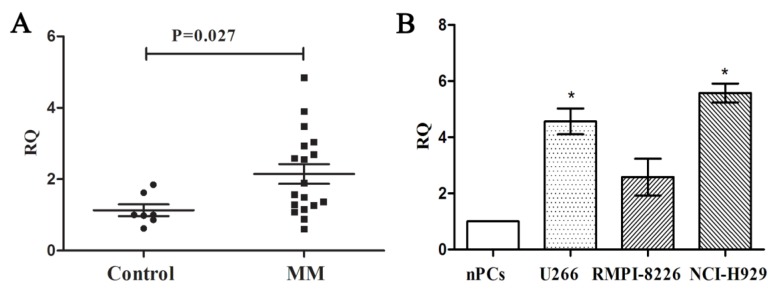
** Detection of PCAT-1 expression in MM samples and cultured cells. (A)** PCAT-1 expression in 18 newly diagnosed MM samples and 7 healthy donors' tissues was detected by real-time PCR. **(B)** PCAT-1 expression in three MM cell lines and normal bone marrow-derived plasma cells (nPCs). All experiments were performed three times independently, mean ± SD, *P < 0.05.

We measured the expression of PCAT-1 in bone marrow samples obtained from 18 MM patients and 7 healthy donors using real-time PCR firstly to explore its clinical significance in MM. Compared with the healthy ones, MM patients were found higher PCAT-1 expression in bone marrow samples (Figure. 1A). Then the MM cell lines, including U266, NCI-H929 and RIMP 8226, were also used to detect PCAT-1 expression with normal bone marrow-derived plasma cells (nPCs) as the normal control. Same with that in MM patients, PCAT-1 expression was also significantly higher in MM cell lines than nPCs (Figure. 1B). Above results indicated PCAT-1 was up-regulated in MM.

### PCAT-1 promotes survival in cultured MM cells

**Figure 2:**
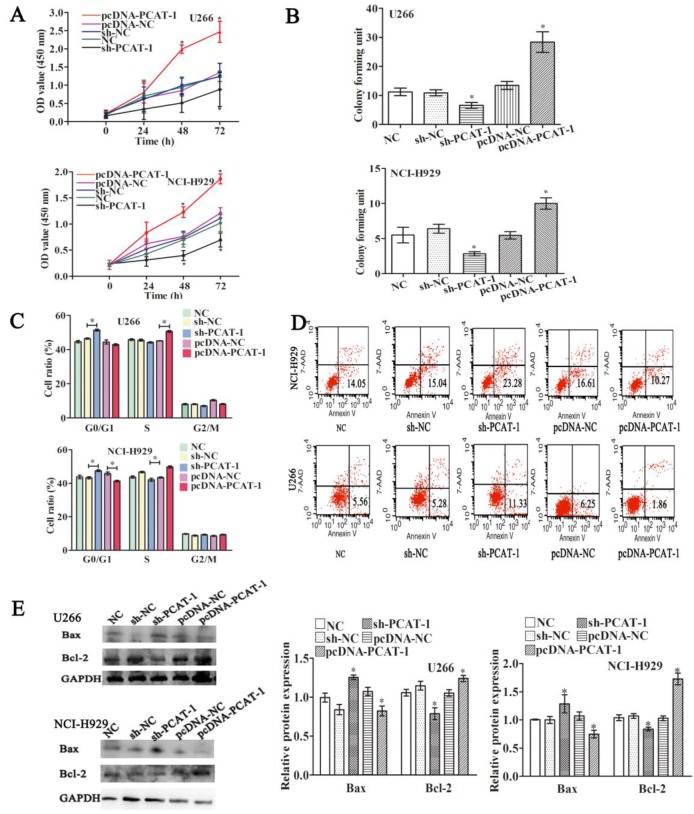
** PCAT-1 facilitated proliferation and survival in cultured MM cells.** NCI-H929 and U266 cells were transfected with the empty vector (pcDNA-NC), PCAT-1 expression vector (pcDNA-PCAT-1), PCAT-1 siRNA (sh-PCAT1) and control (sh-NC). **(A)** Detection of cell proliferation by CCK-8. **(B)** The number of cloned cells as shown by soft agar colony formation assay. **(C)** The examination of cell cycle was obtained from flow cytometric analysis. **(D)** The apoptotic ratio by flow cytometric analysis **(E)** Western blot analysis of apoptosis-related protein Bcl-2/Bax expression. The results were repeated five times independently, mean ± SD, *P<0.05.

To understand its biological functions in MM, PCAT-1 expression was artificially enhanced by transfecting PCAT-1 over-expression vector (pcDNA-PCAT-1) into U266 and NCI-H929 cells, employing the empty pcDNA vector as a negative control (pcDNA-NC). By contrast, PCAT-1-specific shRNA (sh-PCAT-1) was performed to inhibit PCAT-1 expression, using shRNA as a negative control (sh-NC). After ensuring good transfection efficiency, the status of cell viability was checked. CCK-8 assay identified enhanced PCAT-1 expression significantly promoted cell proliferation and repressed PCAT-1 negatively regulated cell proliferation in U266 and NCI-H929 cells (Figure 2A). Colony formation assays demonstrated that transfecting with pcDNA-PCAT-1 obviously increased the number of colonies. On the contrary, the number of colonies was significantly fewer in PCAT-1 knockdown groups than that in controls (Figure 2B). In addition, other tumor phenotypes were also checked. PCAT-1 over-expression decreased the transition from G1 to S phase in the cell cycle assay (Figure 2C). Meanwhile, as shown in Figure 2D, apoptotic cells in pcDNA-PCAT-1 group were significantly less as compared with the control by flow cytometric analysis, while sh-PCAT-1 led to apoptosis promotion in NCI-H929 and U266 cells (Figures 2C-D). Overexpression of PCAT-1 also elevated the level of Bcl-2 protein by around 30% while that of Bax protein was lowered by approximately 23%, as revealed by western blot, and sh-PCAT-1 exhibited the opposite effects (Figure 2E). Taken together, PCAT-1 accelerated division and slowed down apoptosis in MM cells.

### PCAT-1 knockdown increases Bortezomib sensitivity

**Figure 3:**
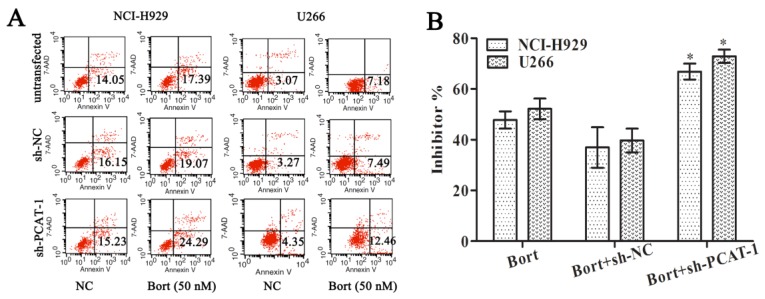
** PCAT-1 knockdown increased Bortezomib sensitivity.** NCI-H929 and U266 cells transfected with or without PCAT-1 siRNA (sh-PCAT1) and control (sh-NC) were treated with 50nM Bort for 48 h. (A) Flow cytometry analysis for apoptotic ratio of MM cells. Data were represented as the independent experiments. (B) WST-1 assay for cell viability determination. Data were summarized from five independent experiments. *P < 0.05.

To access the effect of PCAT-1 on drug sensitivity, MM cells were treated with Bortezomib (Bort) following transfection with sh-PCAT-1. It was surprising to observe that combination treatment with sh-PCAT-1 and Bort in U266 cells generated more apoptosis cells than Bort alone or sh-NC group (12.46% vs. 7.18% and 7.49%, P<0.05). Meanwhile, combination treatment with sh-PCAT-1 and Bort in NCI-H929 cells also exhibited the same high suppression effects with apoptotic rate reaching to 24.29 % in comparison with 17.39% for Bort and 19.07% for sh-PCAT-1 (P<0.05, Figure 3A). The result showed PCAT-1 konckdown strengthened Bort-induced apoptosis in MM. In addition, the synergistic effect of sh-PCAT-1 with Bort was assessed. In Figure 3B, the mitotic suppressive rate of U266 cells in Bort alone group was lower than that in sh-PCAT-1 and Bort combination treatment group (52.17%±7.03% *vs.* 72.90% ±4.53%, P=0.039). A similar result was also observed in NCI-H929 cells with 66.83%±5.41% suppression ratio of Bort and sh-PCAT-1 combination treatment group compared with 47.77%±5.87% for Bort alone (P=0.034). Taken together, the results demonstrated that down-regulating PCAT-1 expression could significantly increase sensitivity of MM cells to Bort.

### PCAT-1 knockdown significantly suppresses tumor growth in mice model

**Figure 4:**
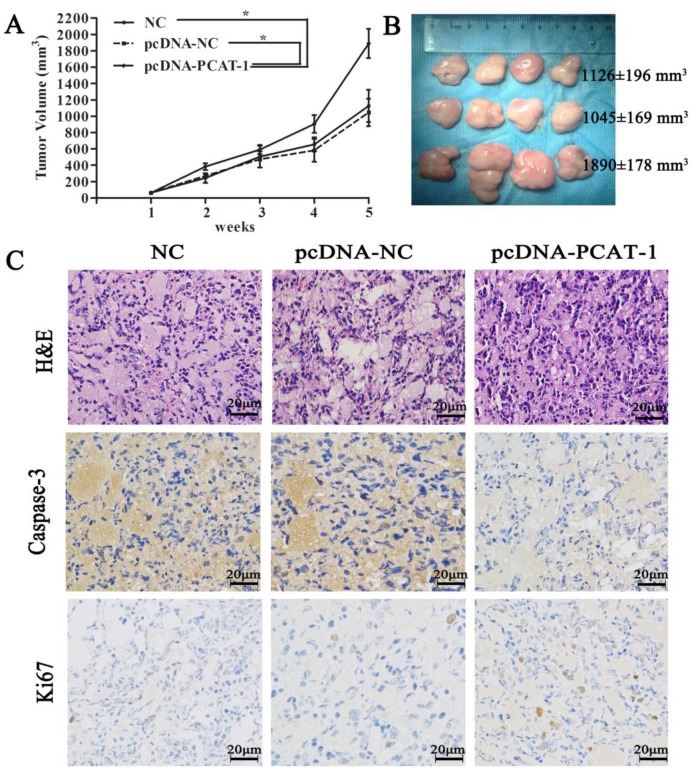
** PCAT-1 inhibition significantly suppressed tumor growth in mice model. (A)** Tumor volume curve was drawn upon the empty vector (pcDNA-NC), PCAT-1 expression vector (pcDNA-PCAT-1) or NC treatment. **(B)** Xenograft tumors excised from mice model were exhibited. **(C)** The specimens were stained with hematoxylin-eosin (H&E). Immunohistochemical staining of caspase-3 and Ki-67 used to assess apoptosis and proliferation (200x). The results were collected from five independent experiments, mean ± SD, *P<0.05.

To discuss the *in vivo* function of PCAT-1, pcDNA-NC- or pcDNA-PCAT-1-transfected U266 cells were inoculated into our animal model respectively. The curves of tumor growth and volume revealed that PCAT-1 overexpression significantly increased the speed and size of xenograft growth in mice. It was found that the tumor volume in NC group, pcDNA-NC and pcDNA-PCAT-1 groups were 1126±196 mm^3^, 1045±169 mm^3^ and 1890±178 mm^3^, respectively. pcDNA-PCAT-1 group owned larger tumor volume than NC and pcDNA-NC groups (Figure 4A-B). Immunohistochemistry of Ki-67 and caspase3 were used for tumor viability testing in xenograft tissues. As shown in Figure 4C, PCAT-1 overexpression decreased the number of cells staining positive for caspase-3 and increased Ki67 staining positive cells, indicating that PCAT-1 could accelerate MM progression *in vivo*.

### GO and KEGG pathway analysis screen PCAT-1-associated signal pathways

**Figure 5:**
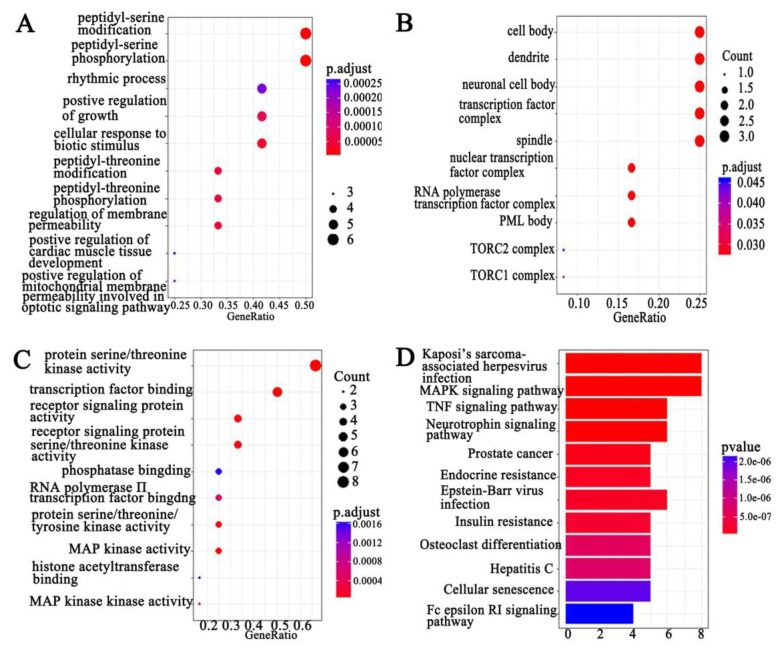
** GO and KEGG pathway analysis.** Functional and signaling pathway analysis of PCAT-1 in MM. **(A)** Biological process. **(B)** Molecular function (MF). **(C)** Cellular component (CC) **(D)** KEGG pathway.

We combined protein chips with Go and KEGG pathway analysis to find out PCAT-1-enriched signal pathways. In terms of analyzing differentially expressed genes, GO and KEGG pathway were performed. Three subtypes were included in GO analysis: cellular component (CC), biological process (BP) and molecular function (MF) (Figure 5A-C). Among differentially expressed genes, up-regulated PCAT-1 were correlated to protein serine/threonine kinase activity, transcription factor binding, receptor signaling protein activity, MAP kinase activity and so on (Figure 5C). Meanwhile, KEGG, a database of gene functions linking genomic information to higher order functional information, indicated the similar results as GO analysis. In detail, the up-regulated genes associated with PCAT-1 overexpression in MM cells were involved in Kaposi's sarcoma-associated herpesvirus infection, MAPK signaling pathway, TNF signaling pathway, Neurotrophin signaling pathway, etc (Figure 5D). To detect differentially expressed proteins (DEP), we analyzed the fold change of each protein individually between two groups. DEPs were defined as those with fold change either over 1.2 or less than 0.83, combining with fluorescent value greater than 150. Finally, 8 DEPs (Table 1) from protein array experiments were selected for validation in the following experiment by western blot.

### PCAT-1 regulates MM cells proliferation and survival via the p38 and JNK MAPK pathways

**Figure 6:**
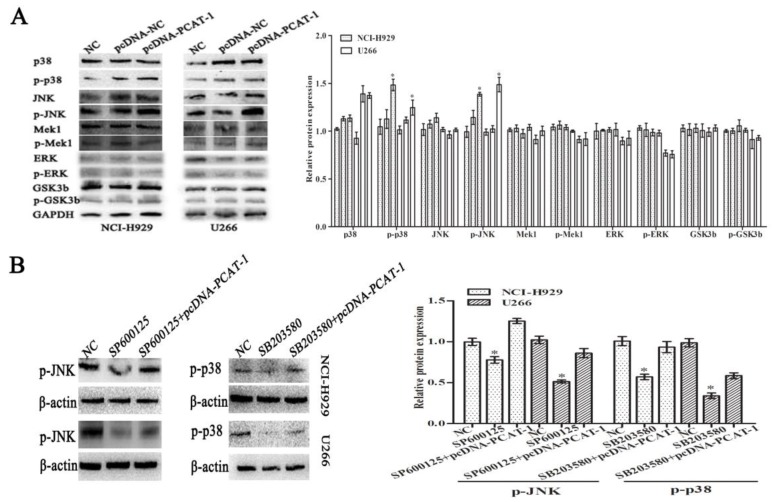
** The p38 and JNK MAPK pathway was involved in PCAT-1-regulated proliferation and survival of MM cells. (A)** NCI-H929 and U266 cells were transfected with the empty vector (pcDNA-NC), PCAT-1 expression vector (pcDNA-PCAT-1) for 48 h. Western blot was performed with MAPK signaling antibodies. The loading amount of protein in each lane was equilibrated by the internal reference GAPDH. **(B)** MM cells transfected with or without PCAT-1 expression vector (pcDNA-PCAT-1) were treated with the specific p38 inhibitor (SB203580) or JNK inhibitor (SP600125). Western blot was performed with MAPK signaling antibodies. All experiments were repeated in triplicate, mean ± SD, *P < 0.05.

To explore the underlying signaling pathways of PCAT-1 in MM, dynamic changes of cell protein was detected by western blot. In pcDNA-PCAT-1 transfection group, the levels of p-p38 and p-JNK were markedly increased, while no significant difference was invented in the levels of p-ERK1/2, p-Mek1 or p-GSK3b (Figure 6A). In order to elucidate the involvement of p38 and JNK in PCAT-1-induced cellular function, MM cells transfected with pcDNA-PCAT-1 were then treated with the specific p38 inhibitor (SB203580) or JNK inhibitor (SP600125). After 24h, we analyzed the changes of pathway-associated proteins by western blot, and found that p38 and JNK MAPK pathways were inactivated by the two inhibitors. More importantly, these inhibitory effects were reversed by PCAT-1 overexpression (Figure 6B), suggesting PCAT-1 promoted MM cells proliferation and survival via p38 and JNK MAPK pathways.

## Discussion

LncRNAs are implicated in several biological functions, including cell proliferation, cell apoptosis, cell cycle control, epigenetic regulation and so on. The abnormal expression of lncRNA is associated with the development and evolution of numerous pathological disorders 14-18. However, the lncRNA expression profile in MM has not been fully elucidated.

Zhou et al 6 identified four-lncRNA signature as prognostic biomarkers, which also predicted overall survival (OS). Shen et al 19 using the GEO database revealed that up-regulated LAMA5-AS1 was a marker to imply better prognosis for MM patients. However, in depth studies are still needed to illuminate whether these lncRNAs can act as molecular biomarkers or what exactly the mechanisms these lncRNA play in MM.

In current study, we demonstrated that PCAT-1 was overexpressed in the patients with newly diagnosed myeloma. Besides, PCAT-1 also exhibited high expression in MM cell lines, indicating PCAT-1 might play a carcinogenesis role in the involvement of MM. The results were consistent with our previous reports, as well as other scholars' findings in another malignancy, such as esophageal cancer 20, osteosarcoma 21-22, cervical cancer 23, and extrahepatic cholangiocarcinoma (ECC) 24. However, PCAT-1 was found to have an opposite expression trend in breast cancer tissues, that was lower PCAT-1 expression in cancerous tissues than in non-cancerous ones 25.

Though several reports of PCAT-1 in malignancy exist, its exact role in MM is still unknown. So we discussed the cellular function of PCAT-1 by applying overexpression or knockdown approaches. Enhanced expression of PCAT-1 could facilitate MM cell proliferation, inhibit apoptosis, and promote drug resistance *in vitro*. Conversely, knockdown of PCAT-1 slowed down the multiplication rate of MM cells, enhanced anti-tumor effects medicated by Bort. Further study demonstrated that combination therapy with PCAT-1 inhibitor and Bort might provide a more effective strategy for MM treatment.

PCAT-1, first identified by Prensner et al 26 in prostate cancer, was found to interact with EZH2 to suppress p21 transcription, which in turn was inhibited by SUZ12 via directly binding to its promoter. Furthermore, PCAT-1 targeted c-Myc to display its functions in prostate cancer 10. Similarly, another study reported PCAT-1 modulated c-Myc and promoted colorectal cancer cells metastasis and multi-drug resistance 27. In this study, we used protein chip arrays, Go and KEGG pathway analysis to explore the potential signaling pathways engaged by PCAT-1. GO and KEGG pathway results showed that PCAT-1 were associated with transcription factor binding, MAP kinase activity, receptor signaling protein activity and so on. Protein chip arrays and western blot validated that the up-regulated genes after transfecting with pcDNA-PCAT-1 in MM cells were involved in MAPK signaling pathway, in which the ERK1/2, JNK and p38 pathways are involved to regulate cell physiology and are also reported to be activated in MM 28-30. Our data indicated that PCAT-1 regulated the survival of MM cells in neither Mek1-dependent nor ERK1/2-dependent manner, but p38- and JNK-dependent. Moreover, the reversal effects of PCAT-1 overexpression were more dominant when MM cells were co-treated with PCAT-1 over-expressed vector and p38 or JNK specific inhibitor, SB203580 or SP600125, respectively.

In conclusion, we highlighted the potential regulatory role of lncRNA PCAT-1 in MM evolution and drug resistance. Further investigation suggested the involvement of p38 and JNK MAPK pathways in molecular mechanisms mediated by PCAT-1. These findings suggested that PCAT-1 may be both a novel potential target and a crucial regulatory factor in MM. Further investigations are necessary to fully clarify the complete potential molecular mechanisms involved in this process.

## Figures and Tables

**Table 1:** The fold change on each protein individually between over-expression of PCAT-1 group and control group.

	meanCtl	meanOE	Fold change
P38	3183.613	6404.642	2.011753
GSK3b	3362.394	5603.721	1.666587
HSP27	728.1473	1019.843	1.4006
P53	2615.33	3560.698	1.361472
JNK	3154.009	4150	1.315786
Erk1/2	2260.291	2823.513	1.249181
Mek1	1347.783	1618.488	0.674954
CREB	2995.776	2022.012	0.674954

## References

[1] Morelli E, Leone E, Cantafio ME (2015). Selective targeting of IRF4 by synthetic microRNA-125b-5p mimics induces anti-multiple myeloma activity *in vitro* and *in vivo*. Leukemia.

[2] Malek E, Kim BG, Driscoll JJ (2016). Identification of Long Non-Coding RNAs Deregulated in Multiple Myeloma Cells Resistant to Proteasome Inhibitors.

[3] Finishing the euchromatic sequence of the human genome (2004). International Human Genome Sequencing Consortium. Nature.

[4] Kapranov P, Cheng J, Dike S (2007). RNA maps reveal new RNA classes and a possible function for pervasive transcription. Science.

[5] Hon CC, Ramilowski JA, Harshbarger J (2017). An atlas of human long non-coding RNAs with accurate 5' ends. Nature.

[6] Zhou M, Zhao H, Wang Z (2015). Identification and validation of potential prognostic lncRNA biomarkers for predicting survival in patients with multiple myeloma. J Exp Clin Cancer Res.

[7] Pan Y, Chen H, Shen X (2018). Serum level of long noncoding RNA H19 as a diagnostic biomarker of multiple myeloma. Clin Chim Acta.

[8] Hu Y, Lin J, Fang H (2018). Targeting the MALAT1/PARP1/LIG3 complex induces DNA damage and apoptosis in multiple myeloma. Leukemia.

[9] Chen L, Hu N, Wang C (2018). Long non-coding RNA CCAT1 promotes multiple myeloma progression by acting as a molecular sponge of miR-181a-5p to modulate HOXA1 expression. Cell Cycle.

[10] Prensner JR, Chen W, Han S (2014). The long non-coding RNA PCAT-1 promotes prostate cancer cell proliferation through cMyc. Neoplasia.

[11] Zhao B, Hou X, Zhan H (2015). Long non-coding RNA PCAT-1 over-expression promotes proliferation and metastasis in non-small cell lung cancer cells. Int J Clin Exp Med.

[12] Yan TH, Yang H, Jiang JH (2015). Prognostic significance of long non-coding RNA PCAT-1 expression in human hepatocellular carcinoma. Int J Clin Exp Pathol.

[13] Shen X, Zhang Y, Wu X (2017). Upregulated lncRNA-PCAT1 is closely related to clinical diagnosis of multiple myeloma as a predictive biomarker in serum. Cancer Biomark.

[14] Prensner JR, Chen W, Iyer MK (2014). PCAT-1, a long noncoding RNA, regulates BRCA2 and controls homologous recombination in cancer. Cancer Res.

[15] Zhao X, Liu Y, Yu S (2017). Long noncoding RNA AWPPH promotes hepatocellular carcinoma progression through YBX1 and serves as a prognostic biomarker. Biochim Biophys Acta Mol Basis Dis.

[16] Hu X, Hong Y, Shang C (2019). Knockdown of long non-coding RNA SNHG5 inhibits malignant cellular phenotypes of glioma via Wnt/CTNNB1 signaling pathway. J Cancer.

[17] Schmitt AM, Chang HY (2016). Long Noncoding RNAs in Cancer Pathways. Cancer Cell.

[18] Wang X, Kan J, Han J (2019). LncRNA SNHG16 Functions as an Oncogene by Sponging MiR-135a and Promotes JAK2/STAT3 Signal Pathway in Gastric Cancer. J Cancer.

[19] Shen Y, Feng Y, Chen H (2018). Focusing on long non-coding RNA dysregulation in newly diagnosed multiple myeloma. Life Sci.

[20] Zhen Q, Gao LN, Wang RF (2018). LncRNA PCAT-1 promotes tumour growth and chemoresistance of oesophageal cancer to cisplatin. Cell Biochem Funct.

[21] Huang J, Deng G, Liu T (2018). Long noncoding RNA PCAT-1 acts as an oncogene in osteosarcoma by reducing p21 levels. Biochem Biophys Res Commun.

[22] Zhang X, Zhang Y, Mao Y (2018). The lncRNA PCAT1 is correlated with poor prognosis and promotes cell proliferation, invasion, migration and EMT in osteosarcoma. Onco Targets Ther.

[23] Ma TT, Zhou LQ, Xia JH (2018). LncRNA PCAT-1 regulates the proliferation, metastasis and invasion of cervical cancer cells. Eur Rev Med Pharmacol Sci.

[24] Zhang F, Wan M, Xu Y (2017). Long noncoding RNA PCAT1 regulates extrahepatic cholangiocarcinoma progression via the Wnt/β-catenin-signaling pathway. Biomed Pharmacother.

[25] Sarrafzadeh S, Geranpayeh L, Ghafouri-Fard S (2017). Expression Analysis of Long Non-Coding PCAT-1 in Breast Cancer. Int J Hematol Oncol Stem Cell Res.

[26] Prensner JR, Iyer MK, Balbin OA (2011). Transcriptome sequencing across a prostate cancer cohort identifies PCAT-1, an unannotated lincRNA implicated in disease progression. Nat Biotechnol.

[27] Qiao L, Liu X, Tang Y (2018). Knockdown of long non-coding RNA prostate cancer-associated ncRNA transcript 1 inhibits multidrug resistance and c-Myc-dependent aggressiveness in colorectal cancer Caco-2 and HT-29 cells. Mol Cell Biochem.

[28] Heuck CJ, Jethava Y, Khan R (2016). Inhibiting MEK in MAPK pathway-activated myeloma. Leukemia.

[29] Wu W, Ma D, Wang P (2016). Potential crosstalk of the interleukin-6-heme oxygenase-1-dependent mechanism involved in resistance to lenalidomide in multiple myeloma cells. FEBS J.

[30] Ohguchi H, Harada T, Sagawa M (2017). KDM6B modulates MAPK pathway mediating multiple myeloma cell growth and survival. Leukemia.

[31] Shen Y, Feng Y, Chen H (2018). Focusing on long non-coding RNA dysregulation in newly diagnosed multiple myeloma. Life Sci.

